# Early detection of osteoporosis in adult children of patients with proximal femur fractures: a real-world screening study

**DOI:** 10.1186/s12891-025-09378-1

**Published:** 2025-12-02

**Authors:** Steffi S.I. Falk, Swenja Block, Thomas Mittlmeier, Hans-Christof Schober

**Affiliations:** 1 Clinic of Trauma, Hand and Reconstructive Surgery, University of Rostock, Schillingallee 35, Rostock, 18057 Germany; 2 OrthoCoast, Practise for Orthopedics and Osteology, Wolgast, Germany

**Keywords:** Osteoporosis, Early detection, Proximal femur fracture, Family history of fracture, Screening, FRAX^®^, Vitamin d deficiency

## Abstract

**Background:**

Osteoporosis is often undiagnosed until fractures occur. A parental history of proximal femur fracture is an independent risk factor for osteoporosis in the next generation. This study evaluated whether adult children of affected patients represent a suitable target group for early screening.

**Methods:**

Over 12 months, patients aged ≥ 70 years with low-energy proximal femur fractures were enrolled. Their adult children were invited to participate. Medical history, laboratory testing, and fracture risk assessment using FRAX^®^ were performed. Treatment thresholds followed AACE guidelines (≥ 3% proximal femur fracture or ≥ 20% major osteoporotic fracture [MOF] risk).

**Results:**

Of 189 patients screened, 183 were eligible (mean age 83 years). Sixty adult children participated (mean age 62 years). One had a prior osteoporosis diagnosis; nine reported previous MOF. Based on FRAX^®^, 40% exceeded treatment thresholds. Vitamin D insufficiency was present in 85%, with supplementation associated with higher serum concentrations.

**Conclusions:**

Adult children of patients with proximal femur fractures represent a high-risk group for osteoporosis. Nearly half met treatment thresholds, supporting targeted screening in individuals ≥ 50 years when a parent sustains such a fracture. This approach may enable earlier diagnosis and prevention of osteoporotic fractures.

## Backround

Osteoporosis is a condition that is characterized by decreased bone mass and impaired bone quality, leading to increased fragility and fracture risk [1]. As a consequence of osteoporotic fractures, patients experience a significant reduction in quality of life, and the disease represents a considerable economic burden. The International Osteoporosis Foundation (IOF) has reported that 5.3 million people in Germany suffer from osteoporosis [2]. Among women over the age of 50, osteoporosis and related fractures are the most prevalent diseases, even surpassing cardiovascular conditions in this population [3].

The first clinical manifestion of osteoporosis is often an so-called indicator fracture. According to the 2023 guidelines of the Dachverband Osteologie (DVO), vertebral body fractures and proximal femur fractures are classified as such indicator events [4]. In Germany, it is estimated that there are 725,000 osteoporosis-related fractures per year. Proximal femur fractures are the most common fractures requiring surgical treatment. They account for 130,000 cases [1].

These data highlight the urgent need to improve diagnostic strategies and identify patients at risk of osteoporosis at an earlier stage. In 2004, Kanis et al. demonstrated that a parental proximal femur fracture is an independent risk factor for osteoporosis in the next generation [5]. This observation could serve as a valuable tool in case finding.

The aim of this study was therefore to investigate whether early diagnosis of osteoporosis is feasible by screening adult children of patients with proximal femur fractures as an indicator of the disease. To the best of our knowledge, these are the first real-world data addressing this question.

## Methods

Over a 12-month period, all patients aged 70 years or older with a low-energy proximal femur fracture were consecutively and prospectively enrolled (hereinafter referred to as “patients”). Eligible fracture types included medial femoral neck fractures and pertrochanteric femoral fractures (classified as AO 31 A1–B3). Low-energy fractures were defined as resulting from falls from standing height, such as a typical trip-and-fall incident. Exclusion criteria were tumor-related pathological fractures and renal insufficiency requiring dialysis.

Patients were interviewed regarding their children, who were subsequently invited to participate in the study. Adult children who agreed to participate (hereinafter referred to as “participants”) underwent detailed medical history assessment to document existing risk factors. Laboratory tests were performed according to the prevailing DVO guidelines [6]. Fracture risk was calculated using FRAX^®^ [7], estimating the ten-year probability of fracture. Following the recommendations of the American Association of Clinical Endocrinologists (AACE), thresholds for defining osteoporosis and recommending pharmacologic therapy were set at ≥ 3% for proximal femur fracture and ≥ 20% for major osteoporotic fractures (MOF; including distal radius, proximal humerus, and vertebral fractures) [8]. All participants received their individual results. Descriptive statistics were performed using Excel 2019.

## Results

A total of 189 patients with proximal femur fractures were screened. and 183 eligible patients were interviewed according to the exclusion criteria. After applying exclusion criteria, 183 were eligible and interviewed. Their mean age was 83 ± 8 years; 137 were women and 48 men. Six patients were excluded due to pathological fractures.

**Fig. 1:**
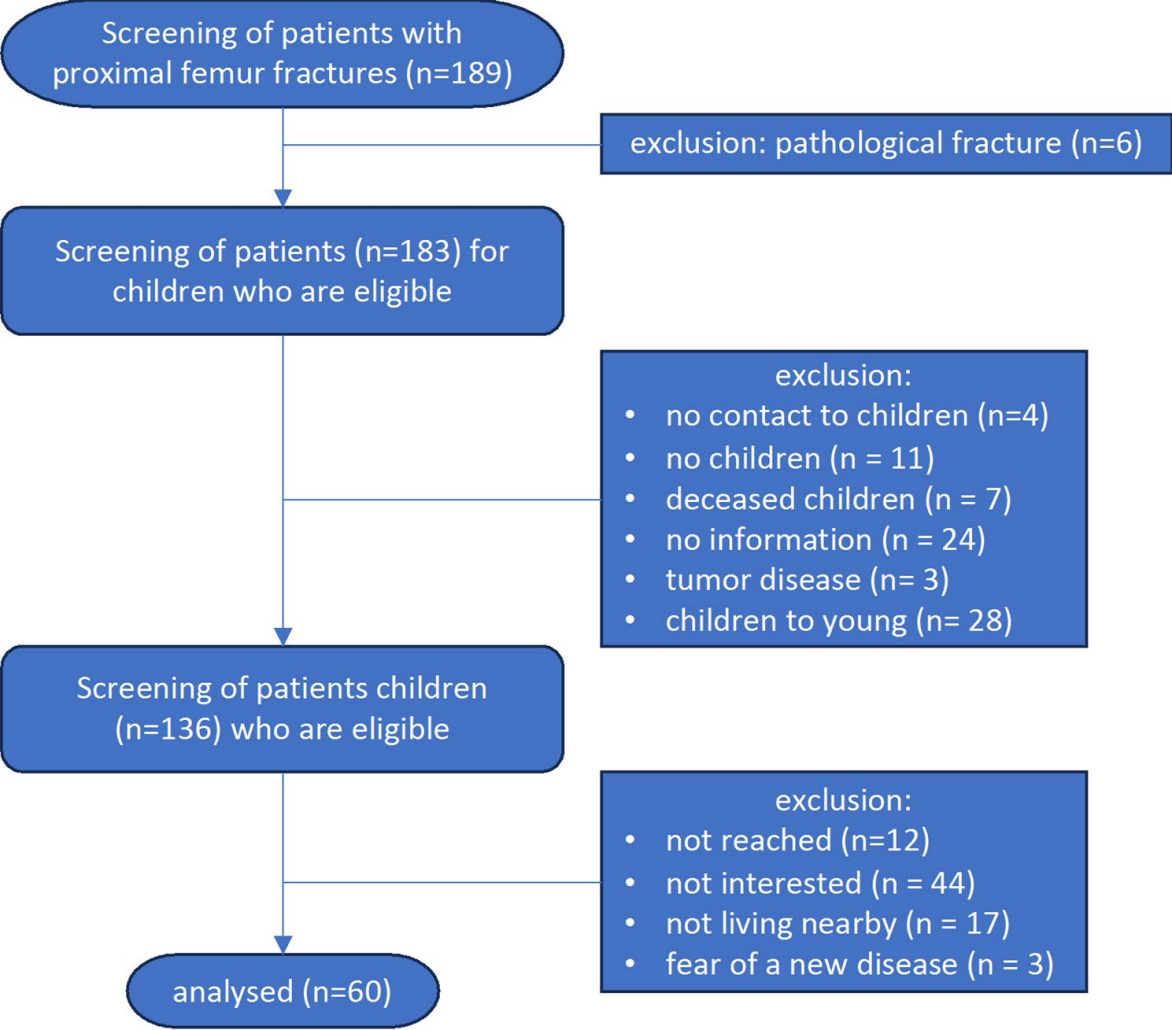
Screening results for the inclusion or exclusion of children of patients with proximal femur fracture and the reasons for rejection

From these patients, 167 children were identified and invited to participate. Major challenges to recruitment included geographic distance between children and parents as well as low interest in additional examinations. Figure 1 provides a detailed overview of patient screening and reasons for exclusion or refusal among the children. Ultimately, 60 participants (38 women, 22 men) were included. Their mean age was 62 ± 6 years (range: 48–73).

Within this group, only one participant had a prior diagnosis of osteoporosis, based on two consecutive distal radius fractures. Nine participants reported a previous MOF (eight distal radius, one proximal humerus fracture).

**Fig. 2:**
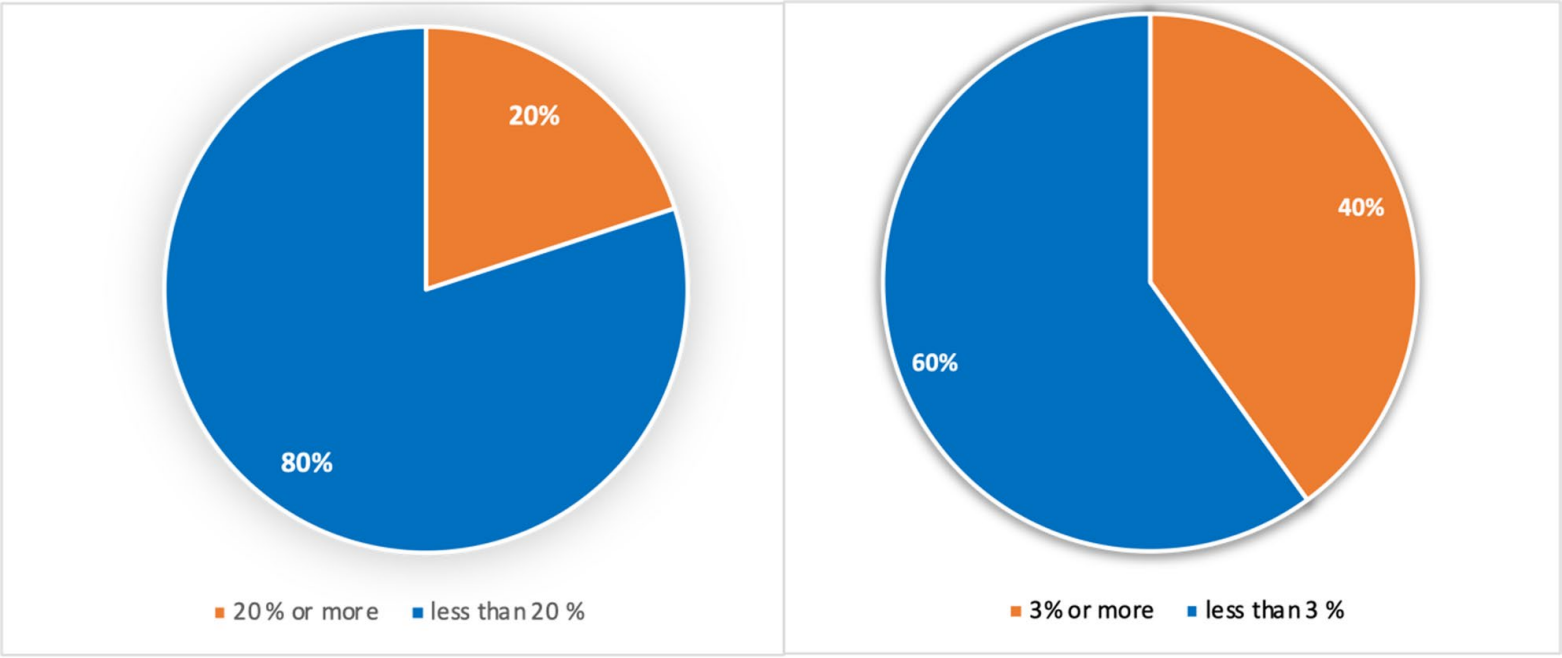
The distribution of participants requiring treatment for a ten-year fracture risk based on the FRAX^®^ risk score, taking into account the AACE threshold, are shown in Figure a for major osteoporotic fractures and Figure b for proximal femur fractures

Based on FRAX^®^ calculations, 12 participants exceeded the 20% risk threshold for MOF, and 24 participants exceeded the 3% threshold for proximal femur fracture within the next ten years (Fig. 2). Thus, 40% of participants were classified as eligible for pharmacologic osteoporosis treatment.

According to the classification of the German Society for Nutritional Medicine [9], 85% of the participants had suboptimal of vitamin D levels. Of the 60 participants, 20 reported intermittent vitamin D supplementation throughout the year. The mean vitamin D concentration among supplement users was 87 ± 34 nmol/l, compared to 50 ± 23 nmol/l in non-users (Table 1).

**Table 1:** Definition of vitamin D status based on serum vitamin levels [9]

values in nmol/l	values in ng/ml	Category	Participants with vitamin D	Participants without vitamin D
< 12	< 4,80	serious deficiency	-	1
12–30	4.80–12	Deficiency	-	5
30–50	12–20	Insufficent	-	14
50–75	20–30	probable insufficent	11	14
75–100	30–40	sufficent optimal	3	5
100–150	40–60	probable too high	5	1
150–375	60–150	hypervitaminosis	-	-
>375	>150	toxic level	-	-

## Discussion

Our pilot study suggests that adult children of patients with proximal femur fractures represent a high-risk group and should be screened for osteoporosis. A proximal femur fracture in a patient over 70 years of age should serve as a clinical trigger for case finding in their offspring.

The presented results indicate that early diagnosis of osteoporosis in this population is feasible. In our cohort, osteoporosis was newly diagnosed in approximately one out of seven screened adult children, and more than one-third of participants met the FRAX^®^ threshold for pharmacologic treatment. To our knowledge, this is the first real-world study to investigate this approach; thus, no comparable datasets are available.

The age and sex distribution of the patient cohort corresponds to the expected epidemiology of proximal hip fractures [10]. Given the high average age of patients, their adult children were typically over 50 years old – already within the range of increased osteoporosis risk. The mean age of our participants was 62 years. Only 28 children identified by patients had to be excluded based on age. These findings support the concept that parental femur fractures identify a subgroup of middle-aged and older adults who should undergo diagnostic evaluation.

The main reason for non-participation was lack of interest. Studies on trial recruitment in oncology suggest that willingness increases when participants anticipate improved treatment, whereas motivation decreases if only diagnostic procedures are offered [11]. Our results align with these observations. Distance to study sites has also been reported as a common barrier to participation [11].

The risk questionnaire and laboratory diagnostics applied in this study adhered to national guideline standards [6]. Therefore, the applied methods were suitable for initial case finding. Based on FRAX^®^ risk assessment, 40% of participants qualified for pharmacologic treatment.

Our results are consistent with the findings of Witzel et al. [12], who confirmed the appropriateness of the 3% treatment threshold for proximal femur fracture risk but suggested that the 20% MOF threshold may be too high, particularly in men. Their recommendation to lower the MOF threshold supports our findings, as a large proportion of participants were identified as requiring therapy.

The observed higher incidence of osteoporosis compared with guideline-based prevalence estimates (15% in women and 2.4% in men aged 50–60 years; increasing to 45% and 17%, respectively, in the subsequent decade [6]) supports parental proximal femur fracture as an independent risk factor and justifies screening in this subgroup.

### Limitations

This study is limited by the relatively small sample size and the 12-month inclusion period. Another limitation is the lack of bone mineral density (BMD) measurements. Instead, the FRAX^®^ score was used as a surrogate. While FRAX^®^ correlates well with BMD and is endorsed by the World Health Organization as a decision-making tool [13], future studies should incorporate dual-energy X-ray absorptiometry (DXA).

## Conclusion

Our data support screening adult children (≥ 50 years) of patients with proximal femur fractures for osteoporosis. This represents the first real-world analysis assessing the feasibility of such screening. We conclude that this approach is both feasible and clinically meaningful.

## Data Availability

The data presented in this study are available upon request from the corresponding author.
